# Sonographic Assessment of Intravascular Fluid Estimate (SAFE) Score by Using Bedside Ultrasound in the Intensive Care Unit

**DOI:** 10.1155/2020/9719751

**Published:** 2020-02-24

**Authors:** Keith Killu, Victor Coba, Dionne Blyden, Semeret Munie, Darlene Dereczyk, Pridvi Kandagatla, Amy Tang

**Affiliations:** ^1^Department of Surgery, Division of Acute Care Surgery, Henry Ford Hospital, Detroit, MI, USA; ^2^Keck School of Medicine, Department of Medicine, Division of Pulmonary, Critical Care, Sleep Medicine, University of Southern California, Los Angeles, CA, USA; ^3^Medical College of Wisconsin, Department of Minimally Invasive and General Surgery, Wauwatosa, WI, USA; ^4^Department of Public Health Sciences, Henry Ford Hospital, Detroit, MI, USA

## Abstract

**Objective:**

The objective of the study was to use an ultrasound-based numerical scoring system for assessment of intravascular fluid estimate (SAFE) and test its validity.

**Methods:**

A prospective, observational study was carried out in the surgical intensive care unit (ICU) of an urban tertiary care teaching hospital. Patient's intravascular volume status was assessed using the standard methods of heart rate, blood pressure, central venous pressure, cardiac output, lactate and saturation of venous oxygen, and others. This was compared with assessment using bedside ultrasound evaluation of the cardiac function, inferior vena cava, lungs, and the internal jugular vein. Applying a numerical scoring system was evaluated by Fisher's exact testing and multinomial logistic model to predict the volume status based on ultrasound scores and the classification accuracy.

**Results:**

61 patients in the ICU were evaluated. 21 (34.4% of total) patients diagnosed with hypovolemia, and their ultrasound volume score was −4 in 14 (66.7%) patients, −3 in 5 (23.8%) patients, and 0 in 2 (9.5%) patients (*p* < 0.001). 18 (29.5% of total) patients diagnosed with euvolemia, and their ultrasound volume score was 0 in 11 (61.1%) patients, +1 in 4 (22.2%) patients, and −1 in 1 (5.6%) patient (*p* < 0.001). 18 (29.5% of total) patients diagnosed with euvolemia, and their ultrasound volume score was 0 in 11 (61.1%) patients, +1 in 4 (22.2%) patients, and −1 in 1 (5.6%) patient (*p* < 0.001). 18 (29.5% of total) patients diagnosed with euvolemia, and their ultrasound volume score was 0 in 11 (61.1%) patients, +1 in 4 (22.2%) patients, and −1 in 1 (5.6%) patient (*p* < 0.001). 18 (29.5% of total) patients diagnosed with euvolemia, and their ultrasound volume score was 0 in 11 (61.1%) patients, +1 in 4 (22.2%) patients, and −1 in 1 (5.6%) patient (

**Conclusion:**

Using the SAFE scoring system to identify the IVV status in critically ill patients significantly correlates with the standard measures. A SAFE score of −4 to −2 more likely represents hypovolemia, −1 to +1 more likely represents euvolemia, and +2 to +4 more likely to be hypervolemia.

## 1. Introduction

The use of bedside ultrasound in the ICU has been expanding over the last two to three decades. The application of POCUS has become the standard of care in many ICUs for diagnostic and therapeutic purposes. The evolution of POCUS started in the Emergency Departments (ED) and has been expanding to other areas especially in the acute care settings where recent trials and studies have expanded its use in the ICUs across the globe. In 1990, the American College of Emergency Physicians (ACEP) published a statement in support of the use of bedside ultrasound by ED physicians that were appropriately trained. This was followed by guidelines and policies by the ACEP that are frequently updated. In 2015 [1], the Society of Critical Care Medicine (SCCM) published guidelines for the use of general and cardiac ultrasound [2, 3].

Throughout the years, most studies have focused on individual organs and systems to evaluate the IVV status, such as examining the right and left heart chambers and contractility [2–4], evaluation of the inferior vena cava (IVC) [5–11] and/or its collapsibility, and the internal jugular vein (IJV) [12–15] and its respiratory variation, as well as lung water volumes and pulmonary edema [16–19]. Through literature review, we failed to identify a scoring system or a numerical value to help standardize different exams. By applying such a system and combining different organs exams, we are hoping to create a more standardized method of ultrasound evaluation of the volume status.

During the past 50 years, ultrasound examination of the heart has been crucial in diagnosis of functional heart status, and echocardiography has become the most used and cost-effective imaging method for the heart [2–4]. Cardiac function evaluation with POCUS has been studied extensively over the years. Evaluation of the heart and determining the EF and stroke volume can help identify the cause of hemodynamic instability and if it is cardiac in origin. Obtaining standard views of the heart is relatively easy to do and can be achieved in most patients [20]. Knowledge of image acquisition, interpretation, and the limitation to certain cardiac views taken into consideration can increase the yield of such exams. Many curriculums have been developed to study the cardiac function for POCUS, and most have been validated and the results reproduced.

Studying the IVC is a very common practice in the ICU to assess the volume status. Evaluating the IVC alone to determine the IVV status does not incorporate other factors affecting the hemodynamic status of the patient [8, 9]. Studying the IVC in mechanically ventilated patients as well as those spontaneously breathing has led to its use in the ICU as a common modality for IVV status [21, 22]. Depending on the IVC only can have limitations [23] and by adding other variables to assess the hemodynamics, the value of IVC interpretation in hemodynamic assessment can be enhanced. IVC has become one of the standard modalities in assessment of fluid status and responsiveness and its use as a guide for fluid therapy [6, 24].

Lung water and the presence of pulmonary edema detected by POCUS have been studied extensively, and different signs to identify different causes of shortness of breath as chronic obstructive pulmonary disease (COPD) and the ability to differentiate it from pulmonary edema are well established [16–19]. Also, the treatment of pulmonary edema and the resolution of certain signs and artifacts are well established in the literature. Association of pulmonary edema with fluid status has to be taken into consideration with special attention to causality, whether it is heart failure or simply fluid overload.

The IJV has limited number of studies, but the interest has been increasing in the last few years [12–15]. Evaluation of the IJV is similar in some ways to the evaluation of the central venous pressure (CVP) with inherit limitations. Including the IJV examination in the assessment of the IVV status is very important and can give an excellent view of the circulatory collapse when it is present as well as ruling it out.

Many protocols have been developed in the ED and ICU regarding the use of POCUS to standardize the application and have an organized manner to evaluate the IVV status. Examples of such protocols are rapid ultrasound for shock and hypotension (RUSH) [25], focused assessment of transthoracic echocardiography (FATE) [26], focused assessment with sonography in trauma (FAST) [27, 28], and the addition to detect pneumothorax in extended FAST (E-FAST), and others. None of these protocols use a numerical value to describe the fluid status of the patient.

In our study, we are trying to quantify the findings of different organs examined. We are performing similar POCUS exams to different organs as done by many protocols, with adding a numerical value which will give the operator a value and a target that can be used for initial and subsequent assessments and comparisons. The sonographic assessment of intravascular fluid estimate (SAFE) score consists of adding a predetermined score for the examination of the heart, lung, IVC, and IJV and having a final score for all combined.

## 2. Methods

This prospective, observational study was performed in the surgical ICU of an urban tertiary care teaching hospital. The study was approved by the Institutional Review Boards of the hospital. Informed consent was not pursued to perform the ultrasound since we use POCUS on daily bases as a routine tool for hemodynamic assessment in the ICU. A convenient sample of 61 patients was enrolled in the study.

The selection was based on the need of the primary treating ICU physician to assess the hemodynamic and the volume status of the patient. Recruitment was based on the presenting symptoms leading the treating ICU physicians to decide if the patient needed volume status assessment and whether the volume status was hypervolemia, euvolemia, or hypovolemia.

Patient's intravascular volume status was assessed using the standard methods of heart rate, blood pressure, central venous pressure, cardiac output, lactate and saturation of venous oxygen, and others. Although these measures could be limited in accuracy and may not give us the exact IVV status, they represent the usual, most practical, and commonly used modalities to assess the IVV in clinical practice.

To create a score for the IVV status, we included the heart, lung, IVC, and IJV. Examining the heart will give us the assessment of the cardiac function if there is hyperkinesia or hypokinesia associated with the IVV status. We examined the lungs to assess lung water and the presence of pulmonary edema or not. Identifying a patient with a history of pulmonary fibrosis or new onset ARDS was also noted. The assessment of the IVC was done to correlate with the volume status where measurement of the IVC diameter was done as well as respiratory variations detected. Assessment of the IJV was on the basis of collapsibility and percentage of respiratory variation.

Having examined all this, we created a score for the individual patient combining all these organs and exams. The results were also compared with the treating physician decision about the IVV using the standard method as a reference.

Our score included the heart, lungs, IVC, and IJV. The score we used is as follows (see Table 1).

(A) Heart: depending on the kinetic function of the heart, we assigned a score for the heart function.
  - Hyperkinetic = −1
  - Normal = 0
  - Hypokinetic = +1
(B) Lungs: assessment of lung water and the presence of pulmonary edema was assigned the following scores.
  - <1 B-lines = −1
  - 1-2 B-lines = 0
  - 3 or more B-lines = +1
(C) IVC: assessment of the IVC for the diameter and the respiratory variation.
  - <2.5 cm in widest diameter and >50% respiratory variation in diameter = −1
  - 1.5–2.5 cm in widest diameter and <50% respiratory variation in diameter = 0
  - >2.5 cm in widest diameter and <50% respiratory variation in diameter = +1
(D) IJV: the assessment of the jugular vein was to evaluate the association with respiratory variation.
  - >40% respiratory variation = −1
  - 20–40% respiratory variation = 0
  - <20% respiratory variation = +1

The scores from all four exams would be added to have a final score (SAFE score) for the IVV status. The total scores will be added and interpreted as follows:

(1) $$\begin{array}{c} \text{SAFE score}=\text{cardiac}\left(-1\text{ to }+1\right)+\text{lung}\left(-1\text{ to }+1\right)+\text{IVC}\left(-1\text{ to }+1\right)+\text{IJV}\left(-1\text{ to }+1\right). \end{array}$$

- A SAFE score of −2 to −4 represents IVV status of hypovolemia. −4 is the optimal result.
- A SAFE score of −1 to +1 represents IVV status of euvolemia. 0 is the optimal result.
- A SAFE score of +2 to +4 represents IVV status of hypervolemia. +4 is the optimal result.

The diagnosis and criteria used for IVV status were based on objective data obtained as shown above and the clinical assessment by the treating ICU physician as heart rate, mean arterial blood pressure (MAP) measured by the arterial line, respiratory rate, as well as invasive monitoring with central venous pressure (CVP) measurements, ScVO2, lactate, and Cardiac Index (CI) measured by the pulse contour analysis method. Although this could be limited in accuracy and may not give us the exact volume IVV status, it is the practical and most commonly used modalities to assess the IVV in practice.

The POCUS was performed by operators who were experienced in ultrasound and use this technology on daily basis to assess their patients. This includes senior staff attending physicians, fellows in training, and advance practice providers. Mostly, the ultrasound examiner was not involved in the management of the ICU patient being included in the study, and the impression of the treating physician and the standard data collected were related to the examiner after the POCUS exam was done so that the decision of ultrasound was not biased by these other factors. No patients were excluded once enrolled, and measurements were completed.

The ultrasound exams and images were obtained and stored using the Zonare One Ultra convertible system (Mindray, North America, Mahwah, NJ, USA) and the Sonosite X-Porte system (FUJIFILM SonoSite, Inc., Bothell, WA, USA).

The prescribed examinations and measurement techniques are listed in Table 1.

**Figure 1:**
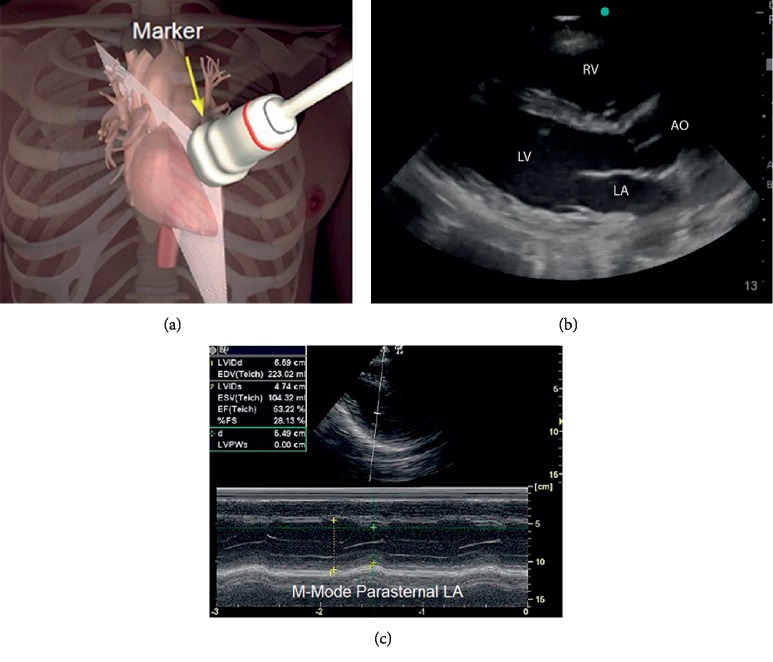
(a) Transducer position on the chest, left parasternal 4-5^th^ intercostal space. (b) Long axis view of the heart. (c) M-mode measuring the ejection fraction (EF).

**Figure 2:**
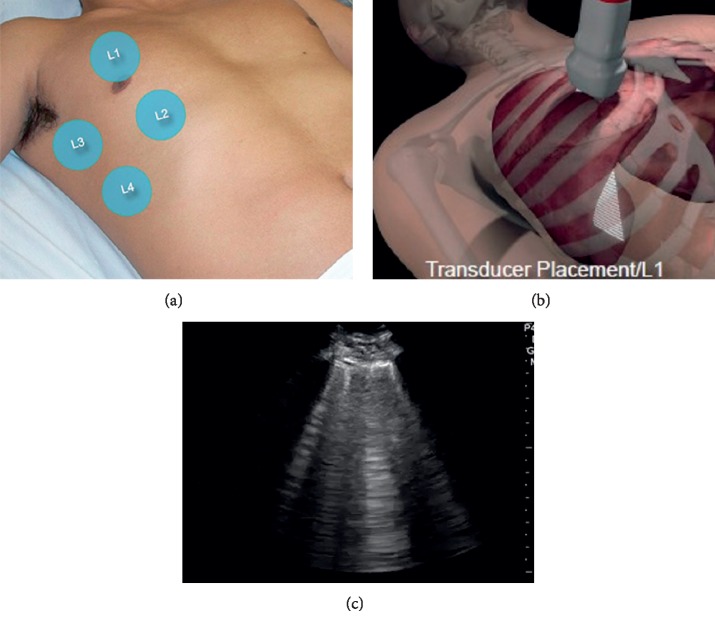
(a) Lung sectors examined: L1 midclavicular line upper chest, L2 midclavicular line lower chest, L3 midaxillary line upper chest, and L4 midaxillary line lower chest. (b) Transducer positioning. (c) Example B-lines.

**Figure 3:**
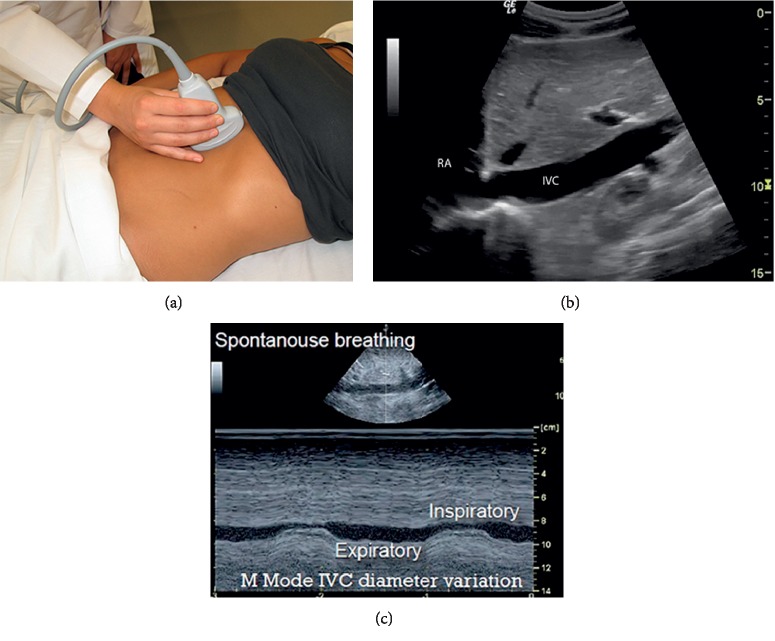
Inferior vena cava (IVC) exam. (a) Transducer placement, midline upper abdomen below the xyphoid. (b) B-mode IVC. (c) M-mode IVC measurements during the respiratory cycle.

The ultrasound exams were done using the standard methods for examining each organ. The cardiac examination was done using a phased array transducer, see Table 1 and Figure 1 for a detailed procedure. The dynamic function of the heart was used as a surrogate for its volume status. The lung examination was done using a phased array or a linear transducer, see Table 1 and Figure 2. The IVC examination was done using a curvilinear transducer for better depth and lateral resolution, see Table 1 and Figure 3. The IVC collapsibility index and respiratory variation were calculated using the following formula:

(2) $$\begin{array}{c} \left[\frac{\left(\text{maximum diameter}-\text{minimum diameter}\right)}{\text{maximum diameter}}\right]\times 100. \end{array}$$

**Figure 4:**
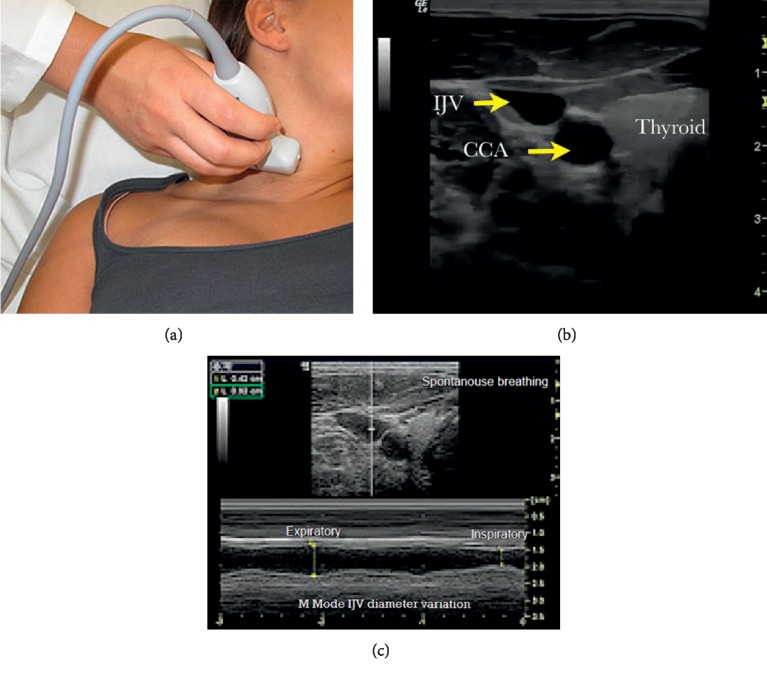
Internal jugular vein (IJV) exam. (a) Placement of the transducer with least or minimal pressure possible. (b) B-mode IJV. (c) M-mode measurement and variation of the IJV during the respiratory cycle. CCA: common carotid artery.

The IJV examination and measurements were done where the head of the bed was placed at a 30-degree angle. The IJV was examined by using a linear transducer and placing it lateral to the level of the cricoid cartilage, see Table 1 and Figure 4. The most circular or maximal diameter was obtained, the degree of variation with respiration was noted, and the collapsibility index was calculated using the following formula:

(3) $$\begin{array}{c} \left[\frac{\left(\text{maximum diameter}-\text{minimum diameter}\right)}{\text{maximum diameter}}\right]\times 100. \end{array}$$

A SAFE score was created by adding the calculated scores for the heart, lung, IVC, and the IJV, and the number ranged from −4 to +4, see Table 1.

## 3. Statistical Analysis

Data were presented as mean ± standard deviation (SD) for continuous variables and frequency (percentage) for categorical variables. Fisher's exact test was performed to evaluate the general associations between the impression of standard measures and the final ultrasound scores. A multinomial logistic model was used to predict the volume status based on ultrasound scores, and the classification accuracy was evaluated. All *p* < 0.05 were considered statistically significant. All analyses were performed using SAS 9.4 (SAS Institute, Cary, NC).

## 4. Results

We evaluated 61 patients admitted to the surgical ICU. The baseline characteristics are listed in Table 2. The mean age was 59 (±14.3) years, 39% were females, and 34% were African American patients. A total of 53 patients (87%) were mechanically ventilated and on positive end-expiratory pressure (PEEP), and 25 patients (41%) were supported hemodynamically by at least one vasopressor. The indications and settings of mechanical ventilation as well as the use of vasopressors or amount of fluids and interventions were all determined by the treating ICU physician. Acute respiratory distress syndrome (ARDS) was present in 6 patients (10%) when the ultrasound examination was conducted.

**Figure 5:**
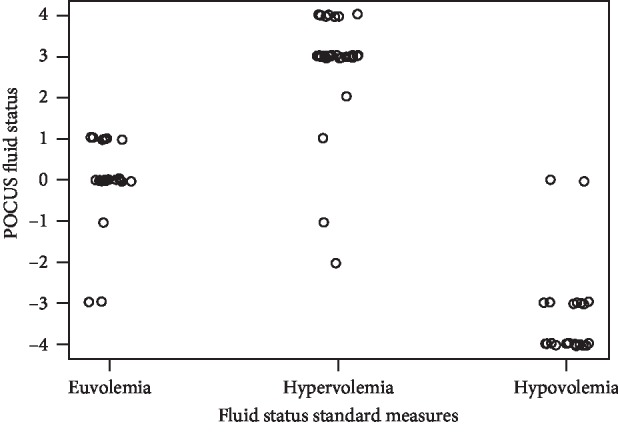
Fluid status estimation with the standard methods compared with the SAFE score. Fisher's exact test, *p* value <0.001, indicating impression standard measures which correlate with the final SAFE scores.

The findings of each exam using the standard measures and correlation with the ultrasound score obtained and the number of patients for each score are described in Table 3. There were 21 (34.4% of total) patients diagnosed with hypovolemia, and the ultrasound volume score in those hypovolemic patients was −4 in 14 (66.7%) patients, −3 in 5 (23.8%) patients, and 0 in 2 (9.5%) patients (*p* < 0.001). There were 18 (29.5% of total) patients diagnosed with euvolemia, and the ultrasound volume score in those euvolemic patients was 0 in 11 (61.1%) patients, +1 in 4 (22.2%) patients, and −1 in 1 (5.6%) patient (*p* < 0.001). There were 22 (36.1% of total) patients diagnosed with hypervolemia, and the ultrasound volume score in those hypervolemic patients was +4 in 4 (18.2%) patients, +3 in 15 (68.2%) patients, and  + 1 in 1 (4.6%) patient (*p* < 0.001). There was a strong association between standard measures and the obtained ultrasound score (*p* < 0.001). SAFE scores of −4 to −2 were more likely to be hypovolemia, −1 to +1 were more likely representing euvolemia, and +2 to +4 were more likely to be hypervolemia. The correct classification rate was 88.5%.  Figure 5 visually shows the separation pattern of standard measures and ultrasound scores.

## 5. Discussion

Using POCUS to assist in determining the IVV status in ICU patients has become a more common practice compared to two or three decades ago and is considered an adjunct to the standard of care methods for assessing the patient's hemodynamic status. Creating a scoring system to identify a most common value to a specific volume status can help standardize the process and reinforce the findings of multiple organ systems examined.

During daily clinical rounds in the ICU, the treating practitioner uses ultrasound to determine the IVV status of a patient. Having this tool helps add to the armamentarium to reach the most accurate diagnosis. Fragmenting the ultrasound exam or using it for one the system or the organ to reach a diagnosis on the volume status is probably a practice that needs improvements. Many ICU practitioners use protocols like the RUSH and FATE [25, 26] to reach a diagnosis about the IVV status and help assess the hemodynamics, yet all these protocols do not use a standard score to identify and assess the severity of the volume status. Using the IVV status scoring system as the SAFE score can help eliminate some of the limitations and help create a more standardized method when discussing the IVV status of a patient.

For example, to identify a patient with a SAFE score of −4 will convey the level of IVV hypovolemia that the patient is in. This will clinically translate to a more urgent and aggressive mode of resuscitation and management. Follow-up ultrasound examinations can help document the patient response to the intervention of giving fluids. If the SAFE score after administering fluid changes from −4 to −1, for example, it will indicate to the treating practitioner that the patient is euvolemic now or responding to the fluid management. This can be done multiple times during the day and on daily basis. A change from a SAFE score of +3 to a score of 0 in 24 hours after diuresis in a fluid overloaded patient indicates the success of the intended diuresis.

Combining all these systems and using the SAFE score will help decrease the limitations that may encounter the treating practitioner when using POCUS. For example, if the patient is hypovolemic but has underlying cardiomyopathy and low EF, using a total score might still help identify hypovolemia if there are no B-lines, and the IVC was collapsing to give a SAFE score of −2.

Limitations to our study were that the sample was small, and larger trials including more patients are needed. We examined only surgical ICU patients, and this scoring system needs to be tested in other scenarios as ED and intraoperative and other ICUs. One of the inherit limitation to our study is the clinical assessment of IVV by the standard methods using the heart rate, blood pressure, CVP, and others which all have limitations at times, and this may influence the results.

Patients who had pleural effusions presented some challenge in addressing the number of B-lines, where we opted to use other lung zones to evaluate for their presence. Patients with ARDS also presented a challenge but were included, and their B-line values were included in the study.

There will still be patients who have underlying dysfunctional hearts or severe pulmonary hypertension affecting the RV function as well as IJV and IVC that can limit the usefulness and the use of the SAFE score. Also, using the functional status of the heart as a surrogate for the volume was a limitation, but it was used because of the ease and ability to do multiple exams daily. These limitations are present for other modalities used to test for IVV status as well. More studies are needed to identify the severity of the score and does it correlate with the standard method.

The strengths of our study were that we examined heterogenous group of patients with multiple different diagnoses. The operators performing the ultrasound exams were staff attending physicians, fellows, and advanced practice providers, so the results are more representative of actual daily clinical practices. The protocol was easy to perform and tasks only few minutes at a time, so this allowed the operators to do multiple assessments throughout the day for the hemodynamic status and observe changes to the interventions done.

Larger studies including larger number of patients and different specialty ICUs are needed to investigate the usefulness and validate the results of this study.

## 6. Conclusion

Using the SAFE scoring system to identify the IVV status in critically ill patients significantly correlates with the standard measures. A SAFE score of −4 to −2 more likely represents hypovolemia, −1 to +1 more likely represents euvolemia, and +2 to +4 more likely to be hypervolemia.

## Figures and Tables

**Table 1:** Steps to follow while performing the ultrasound exam for the heart, lungs, IVC, and IJV. Score assignment with different findings during each exam.

Exam type	Exam method	Score assessment
Echo	(i) Place the patient in a supine position if no contraindications. (ii) Place a phased array transducer at the left sternal border 4-5^th^ intercostal space (Figure 1). (iii) Obtain a long-axis view of the heart, note the cardiac function, and estimate the ejection fraction using the eyeballing method or the M-mode with the maximum systole and diastole measurements. (iv) Obtain a short-axis view of the hear, note the cardiac function, and estimate the ejection fraction. (v) Store the images for review.	Assign a score for the cardiac function as follows: (i) EF > 70%, hyperkinetic = −1 (ii) EF 50–70%, normal = 0 (iii) EF < 50%, hypokinetic = +1

Lung	(i) Place the patient in a supine position if no contraindications. (ii) Place a phased array or linear transducer perpendicular between two ribs in all 4 lung sectors, L1–4 on the right and left (Figure 2). (iii) Count the number of B-lines in each sector. (iv) Store the images for review.	Assign a score for lung water as follows: (i) Add the number of B-lines counted from all segments examined, and then divide by the number of segments examined for the average. (ii) Average < 1 B-lines = −1 (iii) Average 1-2 B-lines = 0 (iv) Average 3 or more B-lines = +1

IVC	(i) Place the patient in a supine position if no contraindications. (ii) Place a phased array or curvilinear transducer midline in the epigastric area to locate the IVC. (iii) Measure the IVC diameter just distal to the right hepatic vein, with the maximal and minimal diameter. (iv) Calculate the collapsibility index: ((maximal diameter − minimal diameter)/maximal diameter) × 100. (v) During spontaneous breathing, the maximal diameter will be during expiration and the minimal during inspiration, and the opposite is true during mechanical ventilation. (vi) Store the images for review.	Assign a score for the IVC as follows: (i) 2.5 cm in diameter and >50% variation in diameter during respiration = −1 (ii) 1.5–2.5 cm in diameter and <50% variation in diameter during respiration = 0 (iii) 2.5 cm in diameter and <50% variation in diameter during respiration = +1

IJV	(i) Place the head of the bed at 30 degrees if no contraindications. (ii) Place a linear transducer across the patient' neck in the area of the cricoid cartilage. Hold the transducer with no pressure applied to the vein. (iii) Obtain the largest diameter image of the IJV. (iv) Measure the maximal and minimal diameter at the largest diameter point and the respiratory variation. (v) Calculate the collapsibility index: ((maximal diameter − minimal diameter)/maximal diameter) × 100. (vi) Store the images for review.	Assign a score for the IJV as follows: (i) 40% respiratory variation = −1 (ii) 20–40% respiratory variation = 0 (iii) 20% respiratory variation = +1

**Table 2:** Patient characteristics.

Variable	Response	All patients (*N* = 61)
Age		59.2 ± 14.3
Sex	Male	37 (61%)
	Female	24 (39%)
Race	White	39 (64%)
	Black	21 (34%)
	Other	1 (2%)
Weight		95 ± 24.7 Kg
Height		171.3 ± 9.6 cm
Body mass index		32 ± 7.8
Heart rate		105.2 ± 17.7
Systolic blood pressure		107.6 ± 21.4
Diastolic blood pressure		57.9 ± 13.2
Mean arterial pressure		73 ± 13.1
Central venous pressure		12.2 ± 6.2
Saturation central venous oxygen (ScVO2)		70.6 ± 10.9
Lactate		3.1 ± 3.5
Cardiac output		6.5 ± 2.3
Cardiac index		3.2 ± 1
Mechanical ventilation	Yes	53 (87%)
	No	36 (59%)
Vasopressors	Yes	25 (41%)
	No	36 (59%)
Acute respiratory distress syndrome	Yes	6 (10%)
	No	55 (90%)
Primary diagnosis		
Sepsis/intraabdominal, pulmonary		25 (40.9%)
Pancreatitis		6 (9.8%)
Pneumonia		13 (21.3%)
Heart failure/fluid overload		3 (4.9%)
Gastrointestinal bleeding		6 (9.8%)
Trauma		8 (13.1%)

**Table 3:** Impression of the intravascular volume status by the standard measures compared with the total ultrasound score. Fisher's exact test, *p* value <0.001, indicating impression standard measures correlate with the final ultrasound scores. Bold scores represent the most in that category.

Impression by standard measures	Total ultrasound score									Total patients
	−4	−3	−2	−1	0	+1	+2	+3	+4	
Hypovolemia	14 **(66.67%)**	5 (23.81%)	0	0	2 (9.52%)	0	0	0	0	21
Euvolemia	0	2 (11.11%)	0	1 (5.56%)	11 **(61.11%)**	4 (22.22%)	0	0	0	18
Hypervolemia	0	0	1 (4.55%)	1 (4.55%)	0	1 (4.55%)	00	15 **(68.18%)**	4 (18.18%)	22
All patients	14 (22.95%)	7 (11.48%)	1 (1.64%)	2 (3.28%)	13 (21.31%)	5 (8.20%)	0	15 (24.59%)	4 (6.56%)	61

## Data Availability

All data collected in this research are available for review.
